# Identification of hypoxic macrophages in glioblastoma: Unveiling therapeutic insights from tumour microenvironment analysis

**DOI:** 10.1002/ctm2.70013

**Published:** 2024-09-19

**Authors:** Zhen Qin, Xiu‐Wu Bian, Yu Shi

**Affiliations:** ^1^ Institute of Pathology and Glioma Medical Research Center Southwest Hospital, Third Military Medical University (Army Medical University) and the Key Laboratory of Tumour Immunopathology The Ministry of Education of China Chongqing P. R. China; ^2^ Yu‐Yue Pathology Scientific Research Center and Jinfeng Laboratory Chongqing P. R. China

**Keywords:** glioblastoma, tumor‐associatedmacrophage (TAM), tumor vascular normalization

## Abstract

Tumor‐associatedmacrophages (TAMs) exhibit remarkable heterogeneity in glioblastoma. Spatially resolved single‐cell transcriptomic studies identified a monocyte‐derived TAM subset localized in the peri‐necrotic niche, driven by hypoxic cues to acquire ahypoxia response signature. These hypoxia‐TAMs destabilize endothelial adherens junctions through adrenomedullin paracrine signaling, promoting the formation of hyperpermeable neovasculature that impedes drug delivery. Blocking adrenomedullin produced by hypoxia‐TAMs restores vascular integrity, increases drug deliveryinto tumors, and provides combinatorial therapeutic benefits. Here we discuss the heterogeneity of TAMs regarding functional states and locations in glioblastomas, and propose future directions for studying the temporospatial dynamics of multifaceted TAM.

**Highlights:**

- Single‐cell omics reveal a functionally and spatially distinct hypoxia‐TAM subset in glioblastoma.
- Adrenomedullin secreted by hypoxia‐TAM destabilizes tumor vasculature and its blockade enhances vessel integrity and drug delivery.

## Graphical abstract

Targeting hypoxia‐TAM‐secreted adrenomedullin for tumor vessel normalization.

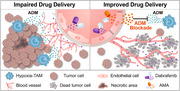

Diffuse gliomas, including isocitrate dehydrogenase (IDH)‐wildtype (wt) glioblastoma (GBM) as well as IDH‐mutant astrocytoma and oligodendroglioma, are the most prevalent and lethal malignant brain tumours in adults.^1^ Tumour‐associated macrophages (TAMs) are abundant in these tumours, interacting directly with malignant cells to fuel tumour progression and create an immune‐suppressive microenvironment.^2^ However, general TAM studies may not accurately recapitulate the full heterogeneity of macrophage populations. The development of single‐cell RNA sequencing (scRNA‐seq), spatial transcriptomic technologies, and time‐resolved single‐cell transcriptomics has revolutionized the study of transcriptional dynamics and cellular interactions across distinct tumour niches.^3^, ^4^, ^5^, ^6^ These advanced methodologies provide powerful tools for comprehensively exploring the diverse populations of tumour‐infiltrating immune cells, including TAMs, in a temporospatial context. Targeting TAMs through suppressing peripheral monocyte/macrophage recruitment or rewiring TAM immunosuppressive signature presents attractive therapeutic approaches. While various TAM‐targeting approaches are being tested in preclinical models of diffuse gliomas and other solid tumors, clinical trials have yet to show compelling efficacy, likely due to the remarkable cellular diversity and plasticity of TAM populations.^7^ To advance the field forward, it will be necessary to better define the spatial localization of TAM subpopulations and whether and how differential zonation within diffuse gliomas contributes to the division of labour among them, which is crucial for the rational design of effective TAM‐targeting therapies.

In a stud recently published in Cancer Cell, we made a comprehensive description of the full heterogeneity of TAMs in diffuse gliomas.^3^ We obtained scRNA‐seq data derived from 51 cases of diffuse gliomas in humans and 12 cases in mouse GBM and dissected the composition and cellular diversity of TAMs derived from peripheral monocytes and those from brain‐resident microglia. In particular, we found that hypoxia‐TAMs, a monocyte‐derived TAM (Mo‐TAM) cluster characterized by the enrichment of hypoxia response signature, were more frequently present in GBM‐IDHwt relative to IDH‐mutant astrocytoma and oligodendroglioma.^3^ As a hallmark of GBM, hypoxia has been identified as a tissue organizer in several recent studies. Greenwald et al. found that human GBM consists of both disorganized and structured regions, with the structured regions displaying a five‐layered organization linked to hypoxia.^4^ Utilizing a sensitive fluorescent UnaG reporter to track tumour hypoxia in mouse models, Sattiraju et al. reported that hypoxic zones in GBM attract and sequester TAMs.^6^ By integrating spatial transcriptomic datasets analyses, we found that the proportion of hypoxia‐TAMs increased along with the hypoxia gradient, and these cells were preferentially located in the peri‐necrotic region, accompanied by abundant microvessels.^3^ These encouraging results support that TAMs may finely orchestrate their functions in response to niche cues including hypoxia and further highlight the importance of dissecting the spatial zonation‐associated TAM heterogeneity.

The molecular details of niche‐steered TAM reprogramming are unfolding. Chen et al. showed that Mo‐TAMs occupy perivascular regions within GBM, which could be suppressed by CCL2 disruption.^8^ We previously reported that TAMs in the perivascular niche secrete abundant pleiotrophin to stimulate glioma stem cell survival and promote GBM growth.^9^ TAMs enriched in hypoxic niches were found to upregulate creatine biosynthesis under hypoxic stress to feed GBM cells for tumour growth.^10^ Moreover, enrichment of hypoxia‐induced TAMs is also associated with the dysfunction of cytotoxic T cells.^11^ Utilizing an in vitro TAM polarization model, we revealed that tumour cell‐derived secreted protein acidic and rich in cysteine and hypoxia‐induced lactate are critical niche factors for inducing hypoxia‐TAM signature.^3^ Moreover, spatially resolved multiomics allows us to analyze cellular interactions in situ, providing a deeper understanding of the microenvironment. A detailed analysis of the spatial characteristics of hypoxia‐TAMs revealed the secreted protein adrenomedullin (ADM) and its receptor as a molecular link between hypoxia‐TAMs and endothelial cells.^3^ ADM overproduced by hypoxia‐TAMs was further found to impair endothelial junctions, and knockout of *Adm* in mouse macrophages decreased tumour vascular permeability and normalized tumour vasculature.^3^

The development of new TAM‐centered immunotherapies opens a promising avenue for the development of alternative anti‐tumour treatment strategies. Over the years, substantial efforts have been focused on targeting TAMs by inhibiting their recruitment and accumulation, blocking their tumour‐supportive polarization, targeting immune checkpoints and regulators, and rewiring their metabolism, aiming to reinvigorate their antitumour functions.^7^ Recent advances have unveiled ANXA1, CCL8, and IL‐1β as potential targets to rewire the immunosuppressive signature of TAMs in tumour‐hypoxic niches.^6^, ^11^ However, a one‐size‐fits‐all therapeutic approach is unlikely to be effective, given the diversity of TAM functions across different tumour types and stages. A recent study by Zhong *et al*. identified an immunoprotective TREM2^+^ TAM subset in GBM which activates adaptive antitumour immunity.^12^ Adeno‐associated virus‐mediated TREM2 overexpression in myeloid cells represses GBM growth.^12^ Meanwhile, analogous populations of TREM2^+^ TAMs in multiple peripheral cancers are generally tumour‐supportive.^13^, ^14^ A comprehensive investigation of TAM heterogenicity and the contribution of distinct TAM subpopulations to each specific tumour type and stage is a prerequisite for the rational design of macrophage‐centred therapies. Comparative analysis of the Mo‐TAM composition across different tumours has also revealed a similar hypoxia‐TAM subset in peripheral malignancies as in GBM.^15^, ^16^ Targeting hypoxia‐TAMs by inhibiting their secreted ADM using the ADM antagonist (AMA) effectively normalizes vascular function and ultimately increases the delivery of an anti‐tumour agent into xenografts, thereby leading to a greater antitumour response (Figure 1). This result supports the potential feasibility of targeting hypoxia‐TAMs by AMA as an adjuvant vascular normalizing agent in a broad spectrum of tumours.

**FIGURE 1:**
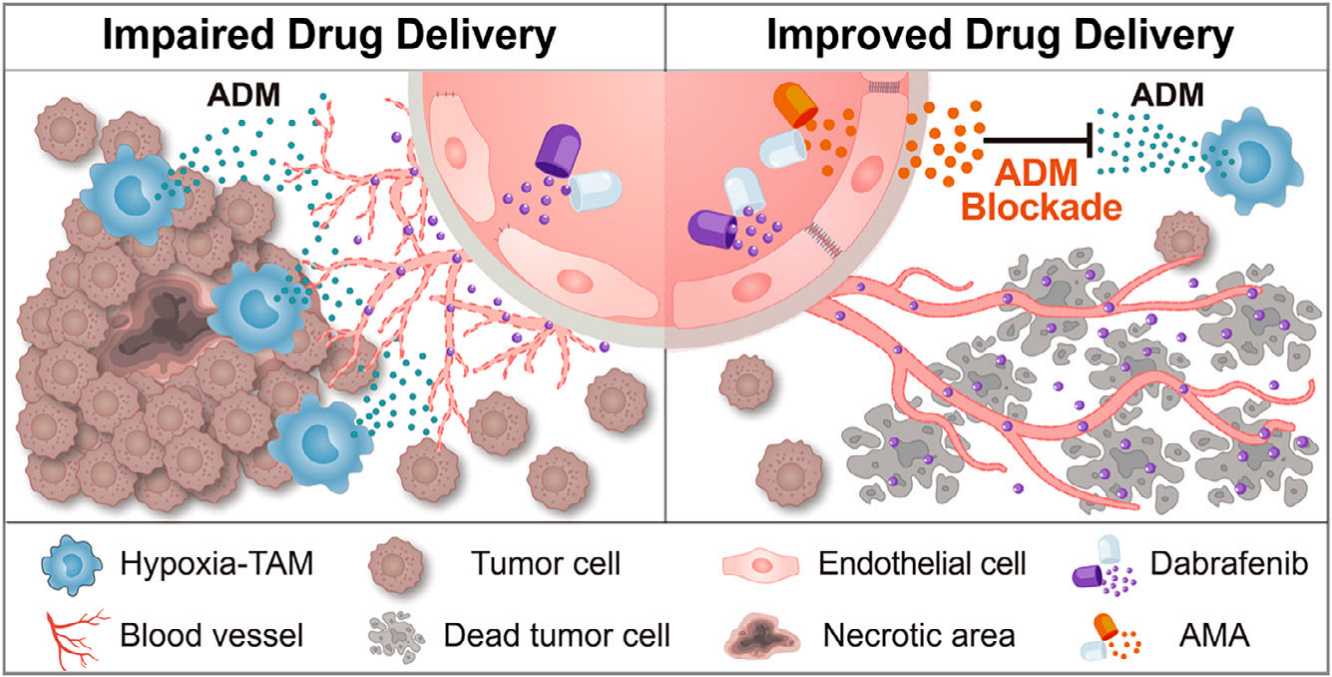
Targeting hypoxia‐TAM‐secreted ADM for tumour vessel normalization. Schematic illustration showing the niche clues from the peri‐necrotic regions induce macrophages to adopt a hypoxic state, termed hypoxia‐TAM, which subsequently augments vascular hyperpermeability. By targeting ADM secreted by this hypoxia‐TAM, tumour blood vessels can be normalized, enhancing the delivery and effectiveness of dabrafenib therapies. ADM, adrenomedullin; AMA, ADM antagonist.

Beyond spatially distinct transcriptional modules, the TAM landscape also evolves during tumour progression and in response to therapeutic interventions. Characterizing TAMs at temporal resolution and capturing their dynamic changes is critically important and currently lacking. More recently, many strategies for achieving lineage tracing at single‐cell resolution, particularly CRISPR‐associated barcoding, have been developed.^17^ Zman‐seq, a single‐cell technology capturing transcriptomic dynamics, integrates fluorescent anti‐CD45 antibodies for in vivo labelling, facilitating empirical measurements of immune dysfunction trajectories in GBM.^5^ By employing immunocompetent GBM mouse models at early and late tumour stages, Sattiraju et al. revealed temporospatial patterning of TAMs coinciding with vascular alterations and the emergence of hypoxic niches.^6^ Characterizing temporal changes in the microenvironments of human tumours is challenging. A recent study on GBM's evolutionary trajectory using rare, multifocal samples offers a promising approach.^11^ Moreover, highlighting time as a protagonist, a recent proposal defines PreTAMs, which are macrophages pre‐existing in the tissue before tumour onset.^18^ Chronic inflammation is designated as responsible for tumour development. PreTAMs, which link inflammation to cancer initiation, will be further reprogrammed within the tumour microenvironment of developing tumours.^19^ By combining time‐stamping with lineage tracing approaches and single‐cell level spatially resolved multiomics, future research will delve into the dynamic changes of macrophages in the tumour development process concomitant with inflammatory stress. Hopefully, the development of new targeted therapies, based on a comprehensive study of TAM heterogeneity using sophisticated methodologies that integrate spatial and temporal information, will pave the way for the development of personalized immunotherapeutic approaches.

## AUTHOR CONTRIBUTIONS

Zhen Qin, Xiu‐Wu Bian and Yu Shi conceived the idea and wrote the manuscript. Xiu‐Wu Bian and Yu Shi revised the manuscript. All authors read and approved the final manuscript.

## CONFLICT OF INTEREST STATEMENT

The authors declare no conflict of interest.

## ETHICS STATEMENT

Not applicable.
